# Standardizing and monitoring the delivery of surgical interventions in randomized clinical trials

**DOI:** 10.1002/bjs.10254

**Published:** 2016-07-27

**Authors:** N. S. Blencowe, N. Mills, J. A. Cook, J. L. Donovan, C. A. Rogers, P. Whiting, J. M. Blazeby

**Affiliations:** ^1^ Bristol Centre for Surgical Research, School of Social and Community MedicineUniversity of BristolBristolUK; ^2^ Clinical Trials and Evaluation Unit, School of Clinical SciencesUniversity of BristolBristolUK; ^3^ Division of Surgery, Head and NeckUniversity Hospitals Bristol NHS Foundation Trust, Bristol Royal InfirmaryBristolUK; ^4^ Centre for Statistics in Medicine, University of OxfordOxfordUK

## Abstract

**Background:**

The complexity of surgical interventions has major implications for the design of RCTs. Trials need to consider how and whether to standardize interventions so that, if successful, they can be implemented in practice. Although guidance exists for standardizing non‐pharmaceutical interventions in RCTs, their application to surgery is unclear. This study reports new methods for standardizing the delivery of surgical interventions in RCTs.

**Methods:**

Descriptions of 160 surgical interventions in existing trial reports and protocols were identified. Initially, ten reports were scrutinized in detail using a modified framework approach for the analysis of qualitative data, which informed the development of a preliminary typology. The typology was amended with iterative sequential application to all interventions. Further testing was undertaken within ongoing multicentre RCTs.

**Results:**

The typology has three parts. Initially, the overall technical purpose of the intervention is described (exploration, resection and/or reconstruction) in order to establish its constituent components and steps. This detailed description of the intervention is then used to establish whether and how each component and step should be standardized, and the standards documented within the trial protocol. Finally, the typology provides a framework for monitoring the agreed intervention standards during the RCT. Pilot testing within ongoing RCTs enabled standardization of the interventions to be agreed, and case report forms developed to capture deviations from these standards.

**Conclusion:**

The typology provides a framework for use during trial design to standardize the delivery of surgical interventions and document these details within protocols. Application of this typology to future RCTs may clarify details of the interventions under evaluation and help successful interventions to be implemented.

## Abstract 2

Design a perfect study

## Introduction

Surgery has recently been recognized as a complex healthcare intervention^1^, ^2^, ^3^, ^4^. Complex interventions comprise multiple interacting components that may be accompanied by concomitant interventions (or co‐interventions), including anaesthesia and elements of preoperative and postoperative care^2^. This complexity can create challenges during the design of surgical interventions in RCTs, in terms of establishing standards of surgery and monitoring whether interventions are delivered as intended. This is exemplified in a recent systematic review^5^ of 80 RCTs, reporting details of 160 surgical interventions, which found that only 47 (29·4 per cent) were reported to be standardized in some way, and monitoring of adherence to the intervention was similarly poor. These issues have partly been addressed through the publication of the SPIRIT^6^ and TIDieR^7^ guidance. The SPIRIT statement provides a checklist of 33 items to be reported in trial protocols. Items relating to interventions (11a–d) recommend that the trial protocol provides information about ‘each group with sufficient detail to allow replication’ and ‘procedures for monitoring adherence to intervention protocols’. The TIDieR guidance – an extension of item 11 of SPIRIT – comprises 12 items relating to the description of all types of intervention, and recommends that the duration, dose and materials used in the intervention are provided.

Although SPIRIT and TIDieR represent important progress for the design and reporting of interventions within RCTs, these guidance documents are not specific for, or easily applicable to, surgical interventions. For example, there is no such thing as a ‘dose’ of a surgical intervention and surgery cannot typically be delivered in an identical manner multiple times. It is difficult, therefore, to know how these recommendations should be used and applied during the design of RCTs in surgery, meaning that the optimal way of describing surgical interventions remains uncertain. The aim of this study was to develop new methods for standardizing and monitoring surgical interventions within RCTs in surgery.

## Methods

### Development of the typology

The typology – defined as a system used for grouping or classifying items according to how they are similar – was informed by a detailed systematic review of how surgical interventions are described, standardized and monitored in published RCTs^5^. Briefly, RCTs in surgery, published between 2010 and 2011, were identified by hand‐searching online archives of the top six journals ranked by impact factor for each of general medicine and surgery^8^. Included were trials in any surgical specialty evaluating a surgical intervention. This was defined as trials that involve physically changing body tissues and organs through manual operation such as cutting, abrading, suturing or the use of lasers^4^. Where available, trial protocols were obtained for each study and analysed in the same way as full‐text articles.

A detailed analysis of the included RCTs and protocols was undertaken using a modified framework approach for the analysis of qualitative data^9^. Framework analysis is usually a deductive approach used to analyse content by assigning descriptive labels, which are outlined *a priori* and collectively comprise a framework. In this study, it was not possible to assign labels *a priori*, and the process was therefore modified such that a subset of ten papers were read and reread to understand the data and develop a preliminary framework. Remaining trial reports were read sequentially and, where required, existing categories were amended or removed, and additional elements added. This iterative process of testing the framework was repeated independently by two researchers until all included papers had been assessed. Once all studies and protocols had been reviewed, a meeting with the research team was held to discuss the proposed typology. Following this, the typology was modified (1 category was added and some existing categories were rephrased). Subsequently, all papers were reassessed with the updated typology to ensure that every description was accounted for.

## Results

### Typology for designing surgical interventions in RCTs

**Figure 1:**
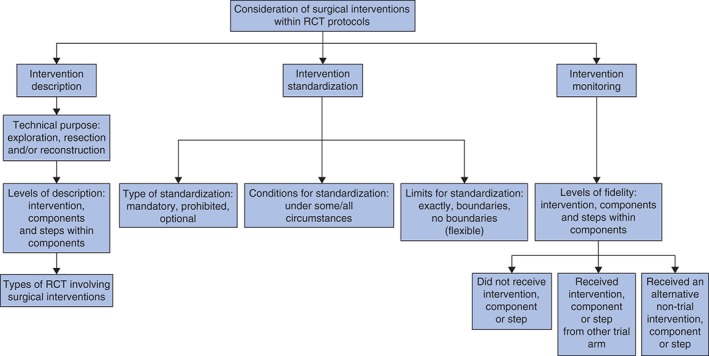
Overview of the typology of surgical interventions

A total of 80 RCTs evaluating 160 interventions within a range of surgical subspecialties were identified (*Fig. S1*, supporting information^10^). Of the 160 interventions, at least some textual description of the surgical intervention (beyond its name) was provided for 118 (73·8 per cent) and this informed the typology. The typology is divided into three sections relating to the description, standardization and monitoring of surgical interventions (*Fig*. 1). The final part of the paper demonstrates how a trial protocol for surgical interventions can be designed using the typology, illustrating this with worked examples.

### Section 1: Intervention description

Three ways of describing a surgical intervention were identified: the overall technical purpose of the intervention, the intervention components and the steps within each component.

#### *Overall technical purpose of the surgical intervention*

The purpose of a surgical intervention can be classified as being exploratory, a resection and/or a reconstruction. These three purposes are not mutually exclusive and some interventions may traverse more than one category. For example, resection of bowel may involve resection (removal of the diseased bowel) and reconstruction (reconnection of the bowel). Initial specification of the overall technical purpose of a surgical intervention facilitates the identification of the underlying components that require consideration.

#### *Identification of intervention components*

Surgical interventions can be divided into components, that is constituent parts or elements of a larger whole. The term ‘component’ was selected (rather than element, part, phase or other similar words) because of its established use in surgical education and training, and within the TIDieR guidance itself 7, 11. A list of all potential components of surgical interventions, identified from the 80 RCTs used to develop the typology, is provided in *Table* 1. Some surgical procedures may include all of the components, whereas others may only include a few. The minimum components of a surgical intervention are the creation and closure of an incision (two components).

**Table 1:** Definitions of the components of surgical interventions

Components of the intervention	Description
Before skin incision	Events associated with the surgical intervention itself, but occurring before the skin incision, e.g. patient positioning, skin preparation, hair removal, surgical scrub
Incision(s) and access	The cut(s) made into skin and deeper tissues. This may require consideration of access, i.e. the method used to approach the operation. Broadly this can be categorized as open or minimally invasive, and further subdivided into multiple‐port, single‐port, robotic or natural‐orifice approaches
Dissection	The process of exposing an organ, tissue or structure
Resection	Removal of all or part of an organ, tissue or structure
Haemostasis	The stopping of bleeding or arrest of blood circulation in an organ, tissue or structure
Reconstruction	The process of rebuilding, repairing or replacing an organ, tissue or structure. This component may include an anastomosis (connection between two structures) or the insertion of a surgical adjunct such as a mesh or prosthesis
Closure	The process of closing or sealing the incision(s). Several layers of closure may be required (e.g. skin, fascia)
After skin closure	Any event associated with the surgical intervention but undertaken after skin closure (e.g. application of dressings or bandages)
Insertion of surgical adjunct	This component relates to the insertion of surgical adjuncts that are not related directly to reconstruction, but are inserted at the time of the surgical procedure (e.g. drains or feeding tubes)
Intraoperative diagnosis	Further characterization of a disease process or anatomy during the surgical procedure itself (e.g. intraoperative cholangiography, blue dye tests or scintigraphy)
Other	Any other component not listed above

#### *Identification of individual steps of interventions*

Detailed analysis of the components of surgical interventions identified that there are steps within each component, representing the precise details within a component. For example, making an incision (1 component) involves several individual details including its location, length, direction and depth. The number and type of steps within any component may be large and wide‐ranging, and vary between interventions. It is therefore not possible to propose a uniform typology for the steps of surgical interventions. Steps can be identified for each intervention once the technical purpose and the constituent components have been established.

It is recommended that descriptions of surgical interventions are considered at three levels in trial protocols: the overall intervention, its components, and steps within each component. Examples are provided in *Table* 2. Initially, establishing all three levels of description of each intervention is necessary. This detailed intervention description can then be used to consider how interventions (and their components and steps) might be standardized (section 2) and monitored (section 3) – if at all – within an RCT.

**Table 2:** Levels of descriptions of surgical interventions

Level of description	Example
Entire intervention	‘The open tension‐free mesh hernioplasty was performed according to Lichtenstein’^12^
Component of intervention	‘Reconstruction consisted of replacement…with an artificial lumbar disc’^13^
Steps within component	‘Pneumoperitoneum was established by open access and maintained at 12–15 mmHg. Three 12‐mm ports were placed: in the midline above the umbilicus, in the epigastrium and in the ipsilateral iliac fossa. A 5‐mm port was placed in the flank’^14^

### Section 2: Standardization of surgical interventions

In an RCT, it is critical to decide whether a surgical intervention needs to be standardized, and how this should be done. Standardization refers to whether the trial protocol specifies exactly how an intervention should be delivered, and may inherently necessitate monitoring during the trial to establish whether centres and surgeons actually followed these instructions. There are several factors that might influence intervention standardization, such as the overall trial design (for example pragmatic *versus* explanatory) or the developmental stage of the intervention^15^. For surgical interventions, it is recommended that three aspects of standardization are considered for each component and step: the type of standardization, conditions relating to it, and the flexibility of delivery. These factors should all be set out clearly in the protocol to inform trial conduct, monitoring and reporting of what was delivered during the trial.

#### *Types of standardization*

The type of standardization required for each component and step of an intervention may be classified as mandated, prohibited or optional. A mandatory step, for example, would be essential to perform in all interventions (and if not performed constitutes a deviation from the protocol), whereas the opposite is true if a step is prohibited. An optional component or step is one that may or may not be performed, at the discretion of each participating surgeon.

#### *Conditions relating to standardization*

During trial design, trialists should identify clinical findings or conditions that may influence the type of standardization required, and detail them in the trial protocol so it is clear what action to take when they are encountered. For example, it may be necessary to decide whether to undertake a cholecystectomy at the same time as a bariatric procedure. A trial protocol therefore needs to describe the conditions relating to this clinical situation: for example, a concomitant cholecystectomy may be mandated *only* among patients with symptomatic gallstone disease (that is, under certain conditions) and prohibited in other patients.

#### *Flexibility of standardization*

A range of flexibility is possible, so that a component or step can be delivered exactly as described within the protocol, within boundaries or totally flexibly. For example, a trial protocol may require surgeons to create an anastomosis using 4·0 polypropylene (exactly as described), any 4·0 or 5·0 monofilament suture (within boundaries) or simply state that this can be performed according to their own preference (totally flexible).

### Section 3: Monitoring of surgical interventions during the trial

Monitoring how surgical interventions are actually delivered in a trial (fidelity) is essential to inform the interpretation of results and subsequent implementation of interventions in practice. Three possibilities for recording and reporting fidelity were identified: the intervention, component or step is not delivered at all; an intervention, component or step from another trial group is delivered instead; or an entirely different intervention, component or step is delivered (*Table* 3). Additionally, the reasons for which the above deviations occur may be crucial and it is therefore recommended that trialists consider recording these throughout the RCT.

**Table 3:** Levels and types of intervention fidelity

Level of fidelity	Types
Deviation from intended intervention	Did not receive any intervention
	Received intervention in other trial arm
	Received an alternative intervention not being evaluated in the trial
Deviation from component(s) of the intended intervention	Did not receive the component
	Component delivered according to description in other trial arm
	Received an alternative component, or component performed in a different way
Deviation from step(s) within component(s) of the intended intervention	Step not done
	Step from other trial arm performed
	Different step performed, or step performed in a different way

### Example of how the typology can be applied to surgical RCTs

The typology was used to design the interventions in two surgical RCTs, and subsequently report these details in the trial protocols. The Rescue‐ASDH (Randomized Evaluation of Surgery with Craniectomy for patients Undergoing Evacuation of Acute SubDural Haematoma) trial^16^ compares the effectiveness and cost‐effectiveness of craniotomy and decompressive craniectomy for acute subdural haematoma. The By‐Band‐Sleeve study^17^ compares the effectiveness of laparoscopic adjustable gastric band, Roux‐en‐Y gastric bypass and laparoscopic sleeve gastrectomy for patients with severe and complex obesity. In conjunction with two of the present researchers and the trial teams, the typology was used to consider the overall purpose of these interventions and to identify the constituent components and steps. Subsequently, the degree of standardization required for each was established. Both are multicentre pragmatic RCTs and all interventions are undertaken routinely within clinical practice. It was therefore agreed that only the key intervention components needed to be standardized, in order to distinguish the interventions in each trial group from one another.

As an example, *Table* 4 lists the components and steps of laparoscopic Roux‐en‐Y gastric bypass (which has a purpose of reconstruction), and the degree of standardization required for each step. The agreed intervention description (as detailed within the trial protocol) is also provided, together with information about fidelity to each aspect of this description. Standardization of the interventions in the Rescue‐ASDH trial (undertaken by surgeons and trialists independent of the typology research team) is described in *Tables S1* and *S2* (supporting information).

**Table 4:** Standardization of laparoscopic Roux‐en‐Y gastric bypass in the By‐Band‐Sleeve study

Components and steps	Laparoscopic Roux‐en‐Y gastric bypass			Description provided in trial protocol^17^	Adherence during trial (*n* = 75)
	Type	Conditions	Flexibility		
Incision and access					
Establishing pneumoperitoneum	Mandatory	None	Veress/open technique	Procedures will be undertaken laparoscopically. Methods used to create a pneumoperitoneum, and the placement of laparoscopic ports and retractors, are at the discretion of the surgeon	75 (100)
Insertion of additional ports	Optional	Poor visibility	Flexible		
Dissection					
Creation of a horizontal pouch	Prohibited	n.a.	n.a.	The pouch can be created according to surgeons' usual practice, although a horizontal gastric pouch that includes fundus is prohibited	75 (100)
Reconstruction					
Measurement of the gastric limb	Mandatory	None	Maximum 150 cm	Methods used to create the biliary and gastric limbs are flexible, although upper limits of 75 and 150 cm respectively are recommended	120 (100–150)*
Measurement of the biliary limb	Mandatory	None	Maximum 75 cm		30 (3–60)*
Opening of the retrocolic window	Optional	None	Flexible	Routing of the Roux limb (antecolic or retrocolic) is flexible	Antecolic 21 (28) Retrocolic 54 (72)
Anastomoses					
Gastrojejunostomy	Mandatory	None	Sutured/stapled, 1–2 layers, oral route or intra‐abdominal	Anastomoses can be performed as the surgeon chooses (e.g. stapled or sutured, circular or linear, single or double layer)	Stapled 75 (100) Circular 10 (13) Linear 65 (87)
Jejunojejunostomy	Mandatory	None	Sutured/stapled, 1–2 layers		Stapled 75 (100) Triple 25 (33) Single 50 (67)
Closure					
Closure of mesenteric defects	Optional	None	Flexible	Closure of mesteric defects is optional	
Peterson's space					59 (79)
Jejunojejunostomy					58 (77)
Mesocolon					54 (100)†
Other					
Use of a bougie	Optional	None	Flexible	Use of a bougie is optional	66 (88)

Values in parentheses are percentages unles indicated otherwise;

* values are median (i.q.r.).

† Only retrocolic reconstructions were included in the denominator, because a mesocolonic window is not created during antecolic bypasses. n.a., Not applicable.

## Discussion

This study describes a novel framework (a typology) for describing surgical interventions in RCTs. It provides guidance on how to consider the extent of intervention standardization in trial protocols, and subsequent monitoring during the trial itself. The typology requires that the overall purpose of an intervention is described, and that it is deconstructed into constituent components and steps. The deconstructed trial intervention then provides a platform to inform the level of standardization of each component and step to be delivered and monitored within the trial. These factors can be discussed and agreed during trial design (potentially as part of pretrial pilot work), so that details for undertaking the surgical interventions can be provided within the main trial protocol, and subsequently monitored during the trial itself. The typology will help to clarify exactly how interventions were intended to be delivered within RCTs and allow the trialists to monitor adherence to this. Application of the typology to RCTs in surgery has the potential to improve trial conduct, and better to inform the implementation of successful interventions in clinical practice.

It may not always be necessary or appropriate to standardize each component or step of a surgical intervention. This should be driven by the research question, the interventions being compared (including the expertise of those delivering them) and whether the trial is predominantly explanatory or pragmatic^15^. In explanatory trials, which determine the efficacy of interventions, great detail may be necessary because the interventions are often novel and their safety needs to be assessed within carefully controlled settings. Pragmatic trials, which determine whether interventions are effective in the real world, are often multicentre studies with large numbers of surgeons. Under such circumstances, specifying each operative step (and those of all accompanying co‐interventions) is likely to create difficulties, and ensuring that each step was delivered as planned may be unrealistic. A balance between adequate standardization and practicality is therefore necessary and appropriate. One way of achieving this is to determine the minimum active ingredients of the intervention^18^ – those that are thought to optimize outcomes or those that are different between the interventions in each trial group – and the degree to which they need to be standardized. In this way, monitoring only the key components may be sufficient, rather than monitoring all components and steps, in order to ensure the intervention is actually delivered as planned.

A potential limitation of this study is that the typology and its categories may not be fully comprehensive. Although further testing could be undertaken with more trial reports, 80 papers were included, providing a total of 160 interventions. The final framework was applied to all papers, and all of the information regarding each intervention could be classified according to the existing typology. Another limitation is that application of the typology was limited to four surgical interventions across two RCTs. A final limitation is that, although specific to surgery, the typology focuses solely on the intervention itself, meaning that, currently, it will need to be used in conjunction with other guidelines such as TIDieR and SPIRIT. Development of a typology for co‐interventions, and identification of the factors that might influence the degree of standardization required (for example explanatory *versus* pragmatic trials), was beyond the scope of this study, which aimed to derive a classification system from existing literature. Work is ongoing in both of these areas, to develop a comprehensive set of guidelines for surgical RCTs. This will require considerable testing, in both new and ongoing studies, across a variety of different interventions and settings, in order to establish its validity and usefulness.

This typology of surgical interventions provides a framework for deconstructing surgical interventions into their constituent components and steps to ensure that all intervention components are considered *a priori*. In a pragmatic trial, after identifying all of the components, those deemed to be key or crucial can be agreed, such that parts requiring standardization are described clearly in the protocol and other components can be delivered according to surgeons' individual preferences. This will allow distinction between mandatory, prohibited and optional steps of an intervention, as well as those that can be delivered flexibly. This approach will require surgeons to agree on a few key details about how an intervention should be performed and within what boundaries, rather than all of its individual steps. Thus, other elements can be undertaken according to personal preference, removing the need for surgeons to conform to a detailed, universal, operative script. More importantly, engaging surgeons in designing interventions in this way may increase the likelihood that they will accept the results of RCTs in surgery and, if interventions are deemed to be effective, actually implement them in routine practice.

**Supporting information**

Additional supporting information may be found in the online version of this article:

**Fig. S1** PRISMA flow diagram of included studies (Word document)

**Table S1** Standardization of surgical interventions in the Rescue‐ASDH trial (Word document)

**Table S2** Final descriptions of Rescue‐ASDH interventions for the trial protocol (Word document)

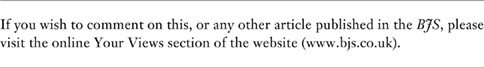


## Supporting information


**Fig. S1** PRISMA flow diagram of included studies
**Table S1** Standardization of surgical interventions in the Rescue‐ASDH trial
**Table S2** Final descriptions of Rescue‐ASDH interventions for the trial protocol
