# Supplementary material for: Standardizing and monitoring the delivery of surgical interventions in randomized clinical trials
Source: Br J Surg. 2016 Jul 27;103(10):1377–84. doi: 10.1002/bjs.10254 (PMC5132147; doi:10.1002/bjs.10254)
Supplement: Supplementary file 1 — Fig. S1 PRISMA flow diagram of included studies Table S1 Standardization of surgical interventions in the Rescue‐ASDH trial Table S2 Final descriptions of Rescue‐ASDH interventions for the trial protocol [file BJS-103-1377-s001.docx]

**BJS10254**

**Methods for standardizing and monitoring the delivery of surgical interventions in randomized clinical trials**

N. S. Blencowe, N. Mills, J. A. Cook, J. L. Donovan, C. A. Rogers, P. Whiting and J. M. Blazeby

**Fig. S1** PRISMA flow diagram of included studies[^10^](#_ENREF_19)

**Identification**

**Initial search results**

n = 4541

Excluded after reviewing abstract

n = 4410

**Screening**

## Full papers accessed

n = 131

**Excluded after reviewing full paper**

n = 51

- Main intervention not surgery
- Primary outcome not reported
- Not an RCT
- More than two trial arms

**Eligibility**

**Included**

**Full papers included in analyses**

n = 80

**Table S1** Standardization of surgical interventions in the Rescue-ASDH trial

| Components and steps | Decompressive craniectomy | | | Craniotomy | | |
| --- | --- | --- | --- | --- | --- | --- |
|  | Type | Conditions | Flexibility | Type | Conditions | Flexibility |
| Before skin incision | Mandatory | None | Flexible | Mandatory | None | Flexible |
| Incision | Mandatory | None | Flexible | Mandatory | None | Flexible |
| Dissection | Mandatory | None | Flexible | Mandatory | None | Flexible |
| Resection  Resection of bone flap | Mandatory | None | Boundaries: must be > 11 cm | Mandatory | None | Boundaries: must be > 11 cm |
| Haemostasis  Evacuation of subdural haematoma  Evacuation of other haematomas | Mandatory  Mandatory | None  If accessible | Flexible  Flexible | Mandatory  Mandatory | None  If accessible | Flexible  Flexible |
| Reconstruction  Replacement of bone flap | Mandatory | None | Boundaries: using screws and plates or titanium device | Prohibited | n.a. | n.a. |
| Closure  Closure of dura    Closure of other layers | Mandatory  Mandatory | None  None | Boundaries: either left widely open or non-constricting duroplasty  Flexible | Mandatory  Mandatory | None  None | Boundaries: either left widely open or a non-constricting duroplasty  Flexible |
| After closure  Dressings | Optional | None | Boundaries: any type except compressive | Optional | None | Boundaries: any type except compressive |
| Insertion of surgical adjunct  Drain  Intracranial monitoring | Optional  Optional | None  None | Boundaries: if drain placed, suction must not be used  Flexible | Optional  Optional | None  None | Boundaries: if drain placed, suction must not be used  Flexible |
| Intraoperative diagnosis | Optional | None | Flexible | Optional | None | Flexible |

**Table S2** Final descriptions of Rescue-ASDH interventions for the trial protocol[^16^](#_ENREF_12)

| **Craniotomy**  The size and location of the incision will be chosen by individual surgeons. A bone flap of at least 11 cm (AP diameter) must be raised. Techniques used to evacuate the subdural haematoma are flexible and surgeons may evacuate other associated haematomas at their discretion. The bone flap must be replaced, either with screws and plates or a titanium device; floating or hinged flaps are prohibited. Dural reconstruction is optional and closure techniques for other tissue layers are flexible. The use of galeal drains and intracranial monitoring devices is discretionary; however, suction drains should be avoided. Compressive head bandages are prohibited |
| --- |
| **Decompressive craniectomy**  The size and location of the incision will be chosen by individual surgeons. A bone flap of at least 11 cm (AP diameter) must be raised. Techniques used to evacuate the subdural haematoma are flexible and surgeons may evacuate other associated haematomas at their discretion. Replacement of bone flaps is not permitted. The dura should either be left open or a non-constricting duroplasty undertaken, according to surgeon preference. Closure techniques for other tissue layers are flexible. The use of galeal drains and intracranial monitoring devices is discretionary; however, suction drains should be avoided. Compressive head bandages are prohibited |
| Measurement of fidelity (bone flap size and replacement or not of the bone flap in both groups) to these steps will be undertaken using postoperative CT |

AP, anteroposterior.
